# Supplementary material for: Deep representation learning of electrocardiogram reveals biological insights in cardiac phenotypes and cardiovascular diseases
Source: iScience. 2025 Jul 28;28(8):113226. doi: 10.1016/j.isci.2025.113226 (PMC12356350; doi:10.1016/j.isci.2025.113226)
Supplement: Document S1. Figures S1–S11, Tables S2–S6 and S9 [file mmc1.pdf]

iScience, Volume *28*

Supplemental information

Deep representation learning of electrocardiogram reveals biological insights in cardiac phenotypes and cardiovascular diseases

Ming Wai Yeung, Rutger R. van de Leur, Jan Walter Benjamins, Melle B. Vessies, Bram Ruijsink, Esther Puyol-Antón, J. Peter van Tintelen, Niek Verweij, René van Es, and Pim van der Harst

Figure S1. Distribution of the latent factors by age and sex.

2D kernel density plots of all latent factors stratified by age (y-axis) in sex subgroups. Purple: women; Green: men.

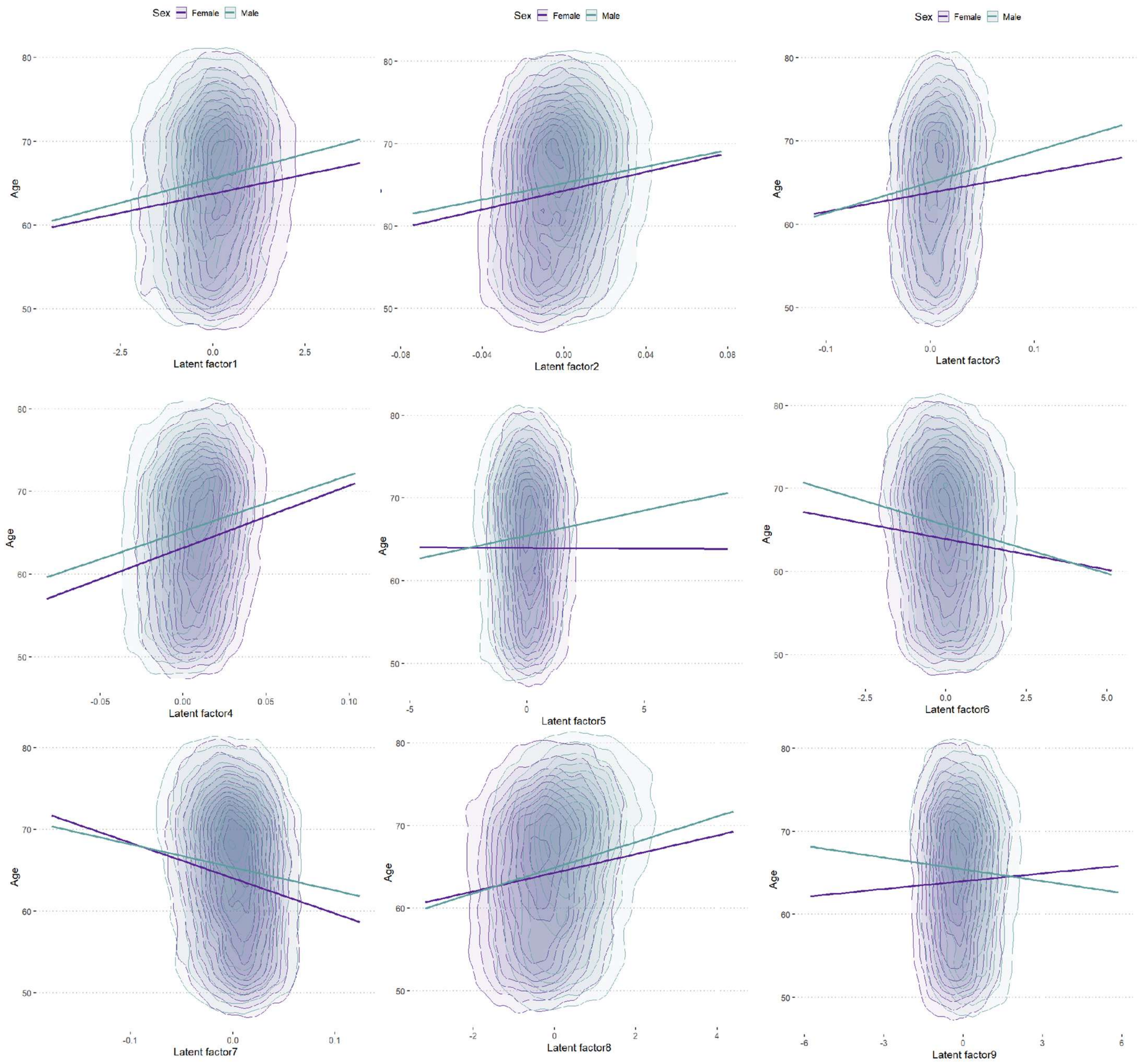

Figure S1 – continued. Distribution of the latent factors 10- 18

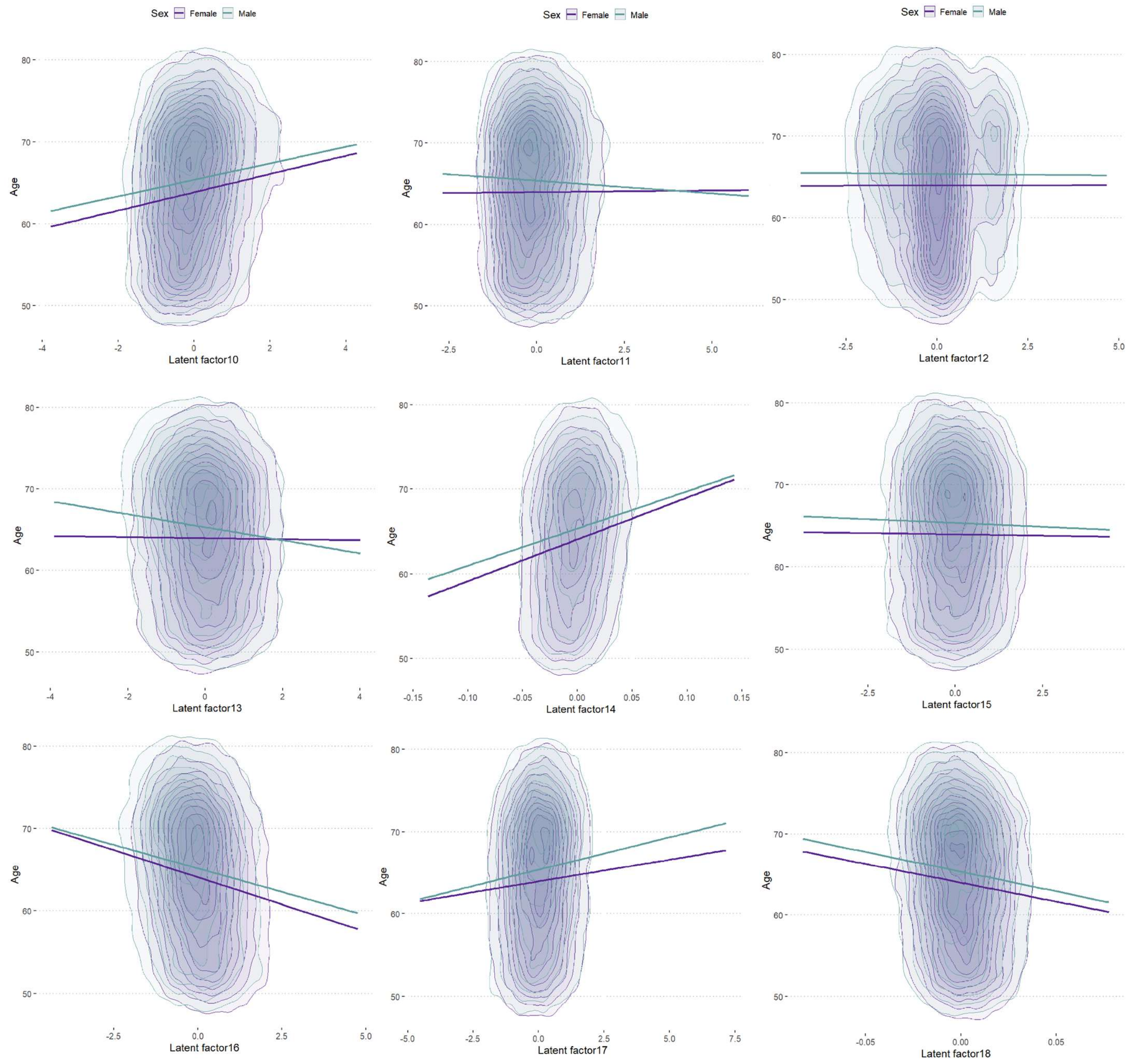

Figure S1 – continued. Distribution of the latent factors 19- 27

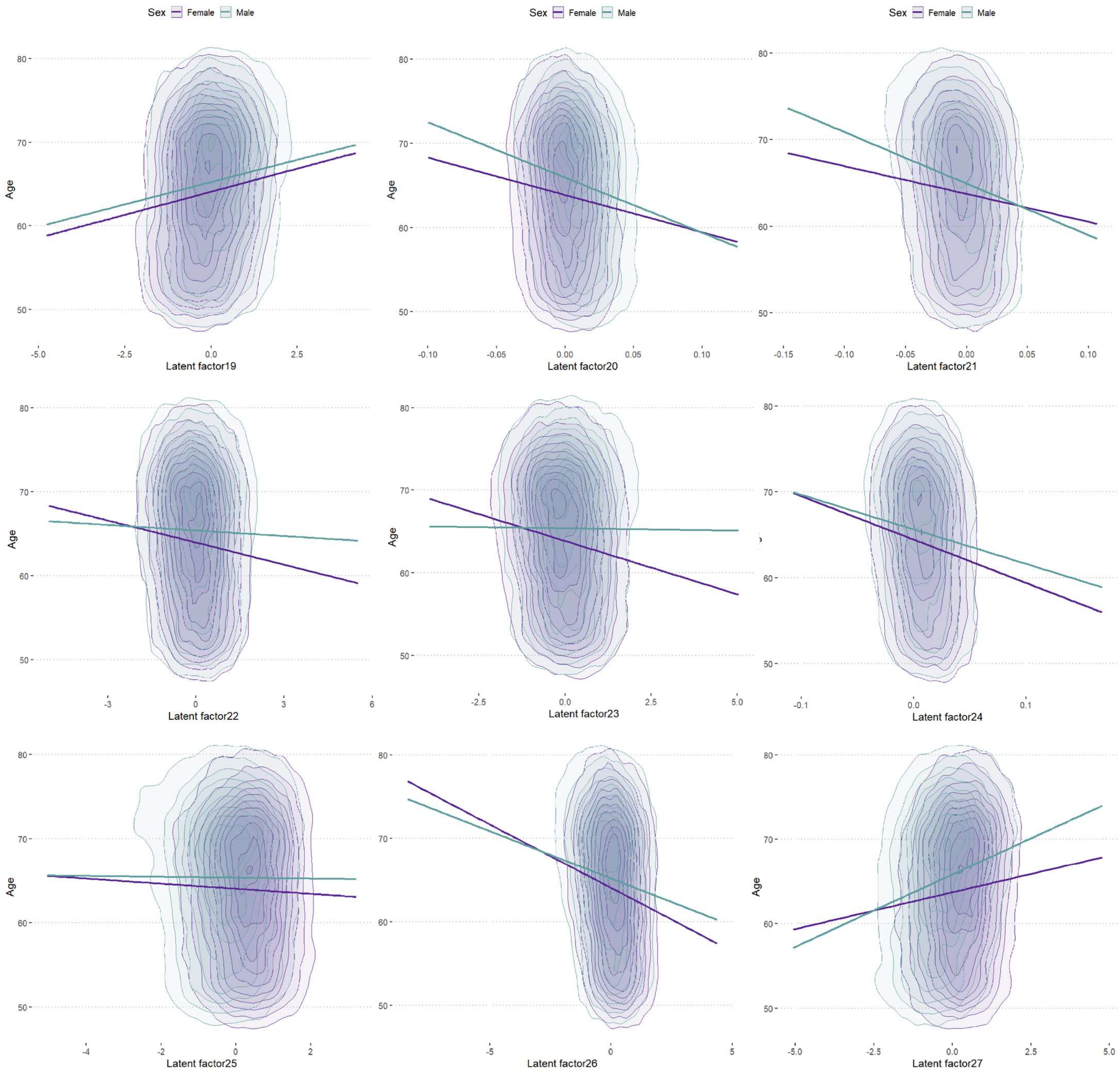

Figure S1 – continued. Distribution of the latent factors 28-32

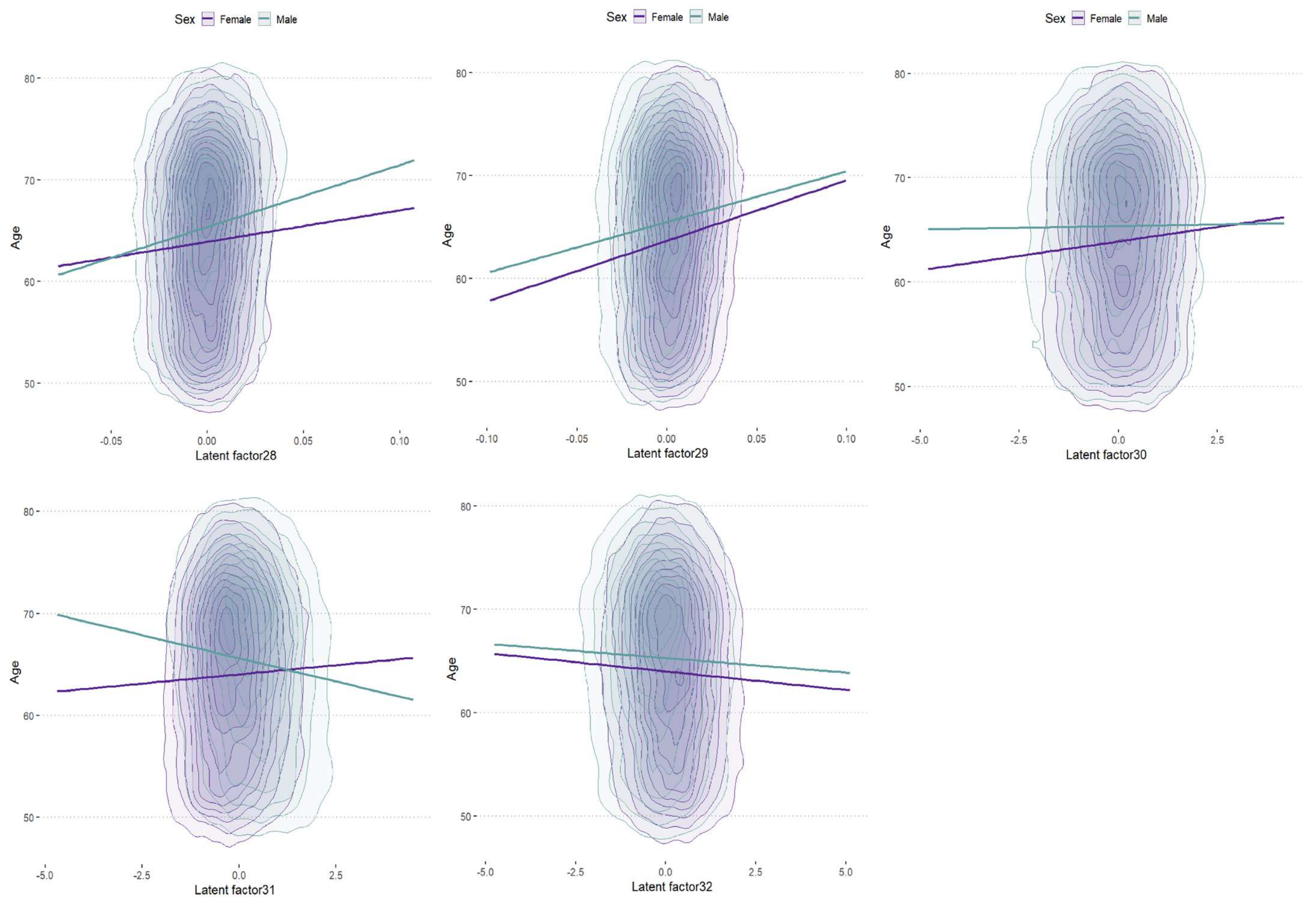

Figure S2 Factor traversals for visualizing the effect of individual ECG factors on the ECG morphology

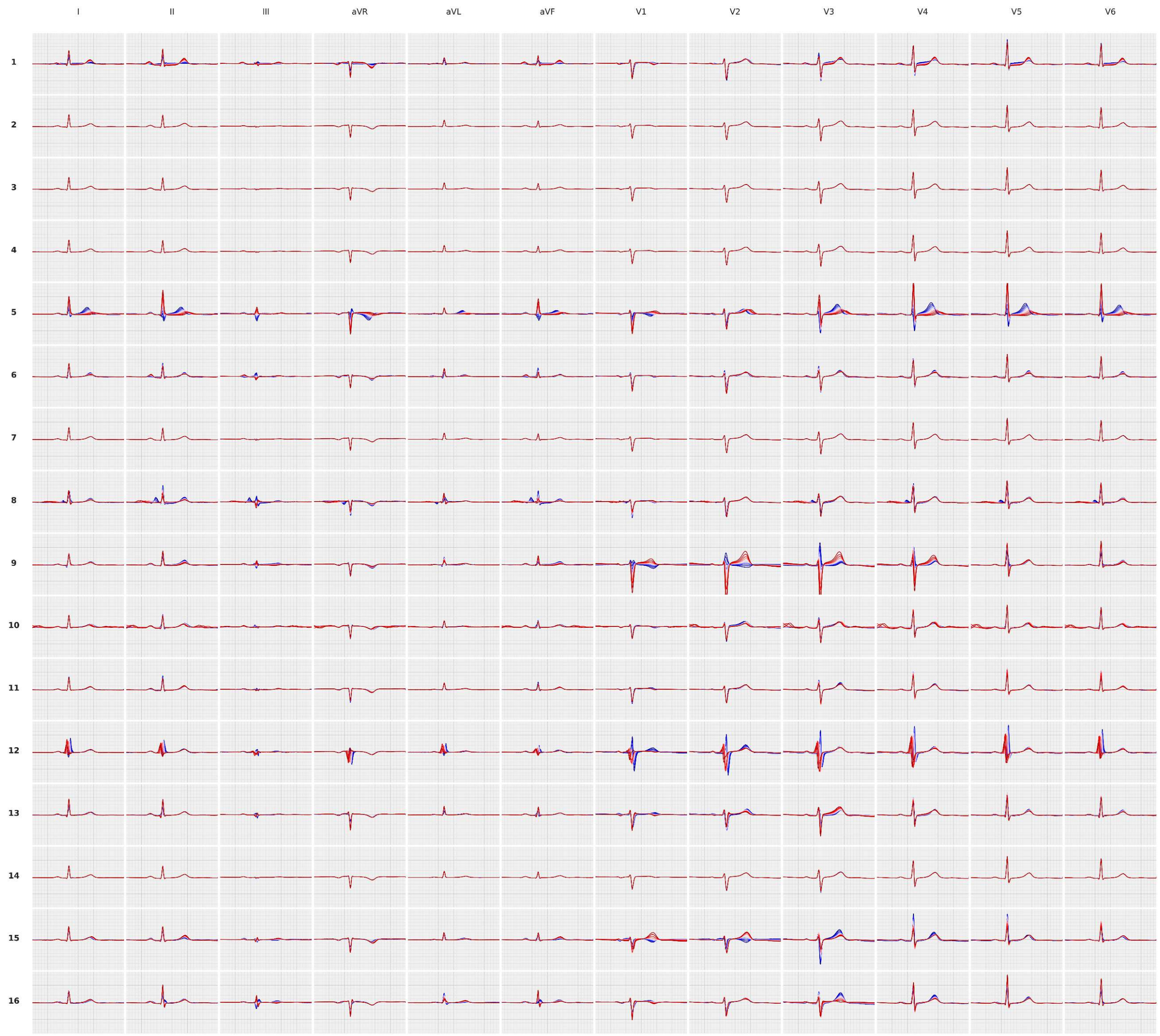

Figure S2 - continued

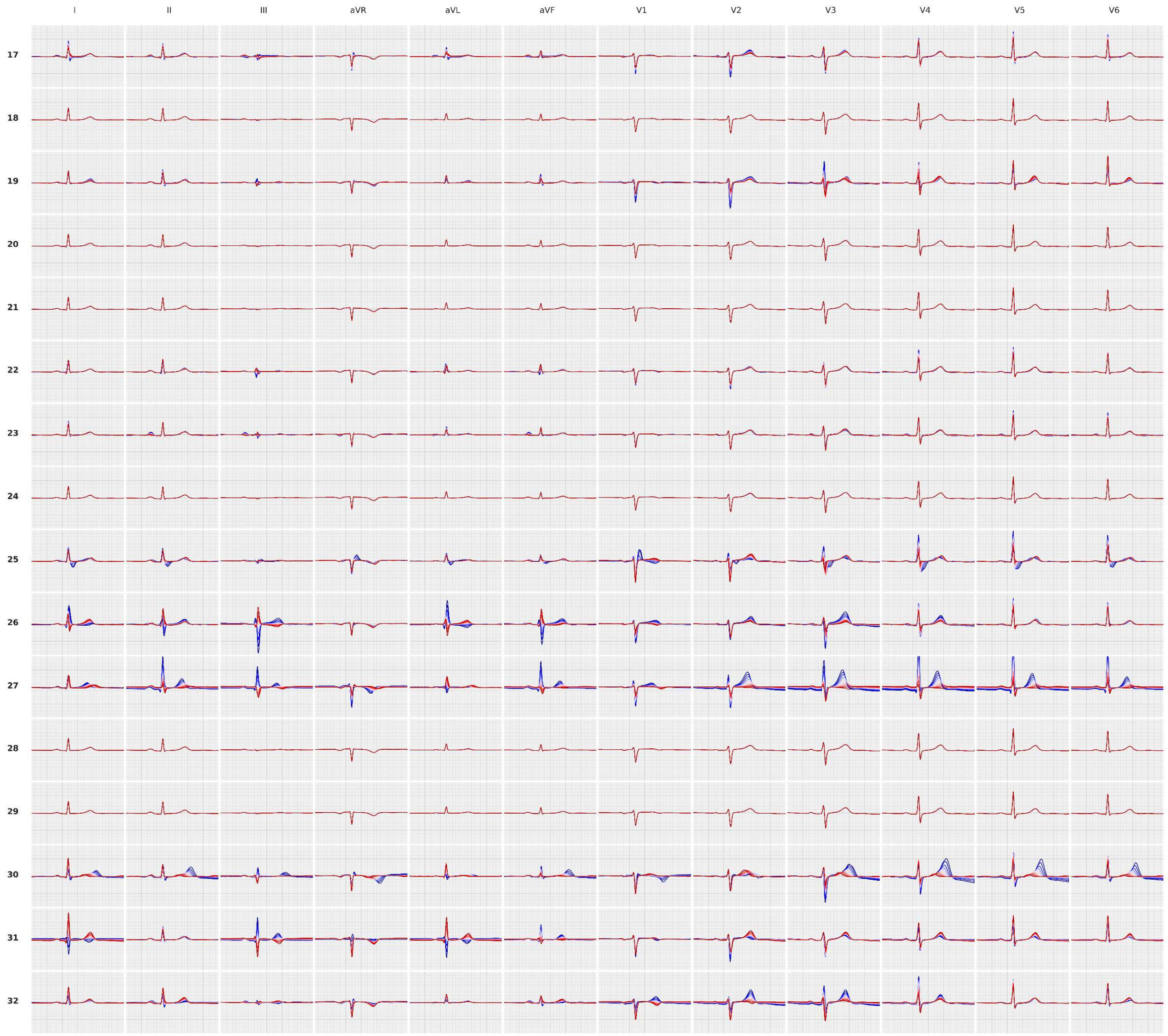

Figure S3 Phenotypic and genetic correlations between the latent factors.

Top right: Pearson correlations between the latent factors; Bottom left: genetic correlations estimated using LD-score regression for latent factors with GWAS summary statistics passing quality control.

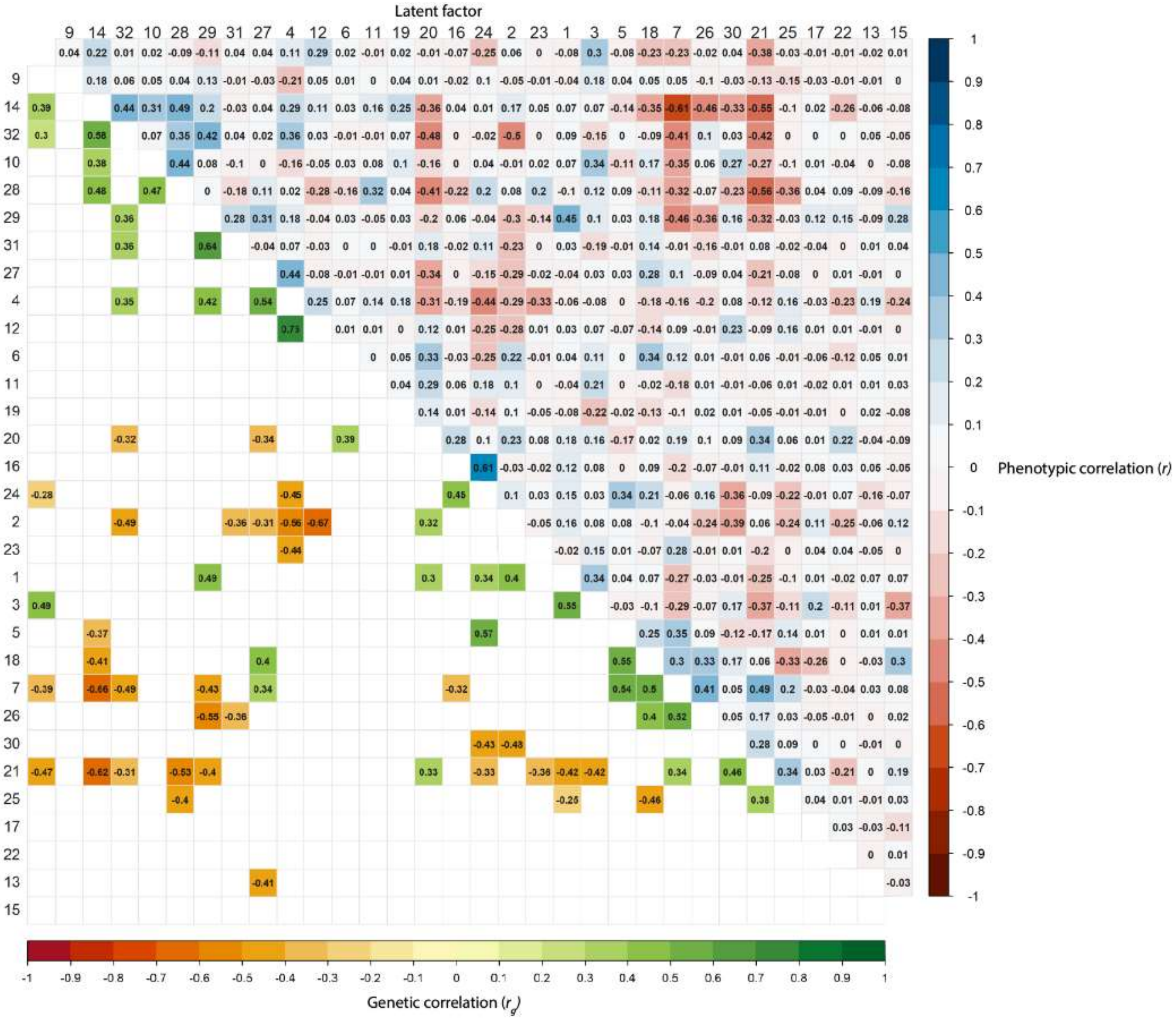

Figure S4. Association of ECG latent factors with additional CMR parameters
Regression coefficients of the latent factors in the multivariate linear regression on each CMR-derived structural and functional parameter. The coefficient describes the effect per standard deviation change of the latent factor. Adjusted R^2 indicates the goodness-of-fit of the linear regression model.

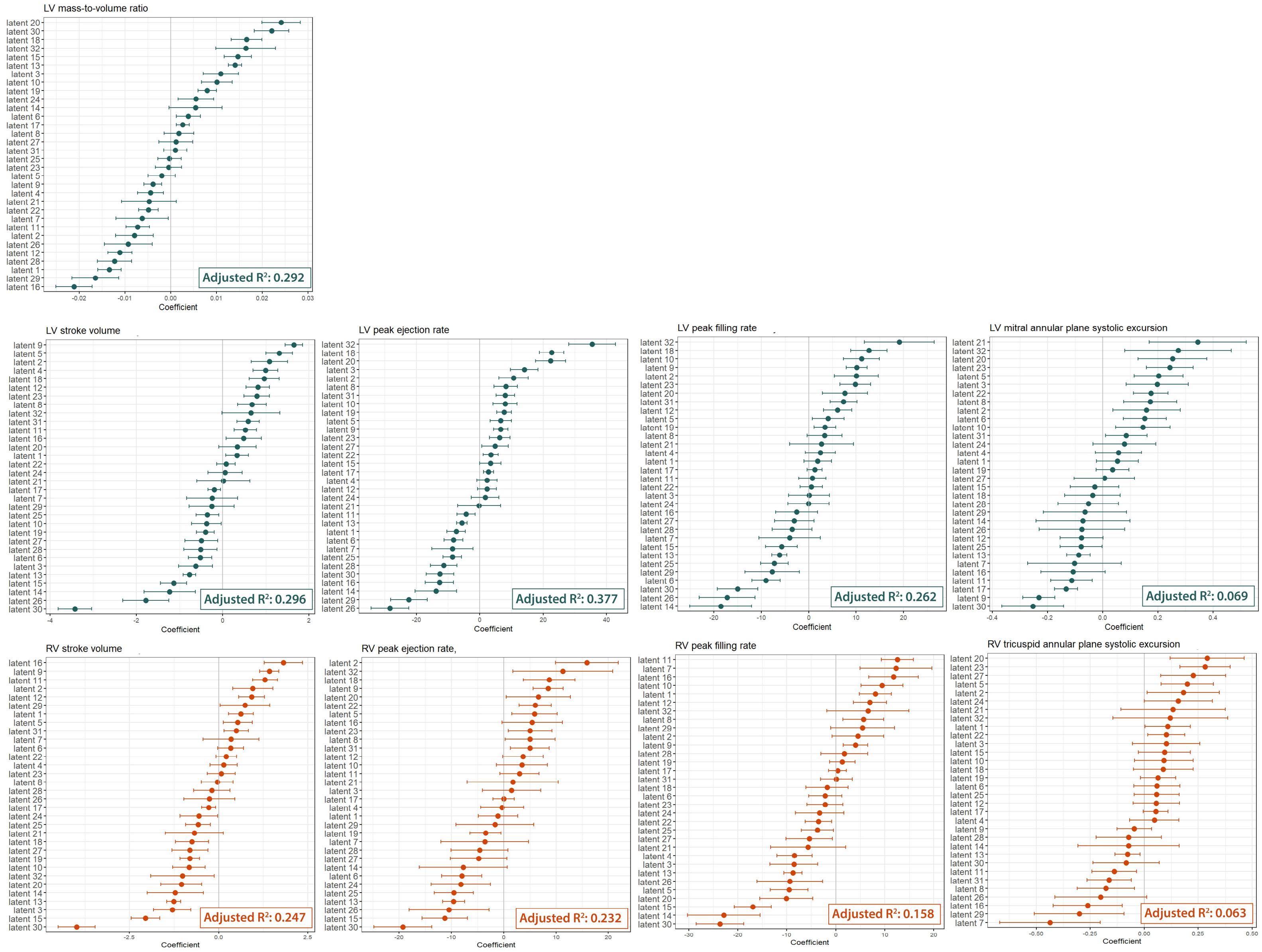

Figure S5 Forest plot for cardiovascular mortality predicted by latent factors

Cardiovascular mortality prediction by the latent factors in cox regressions adjusting for age and sex. Hazard ratio (HR) per standard deviation change of the latent factor is shown.

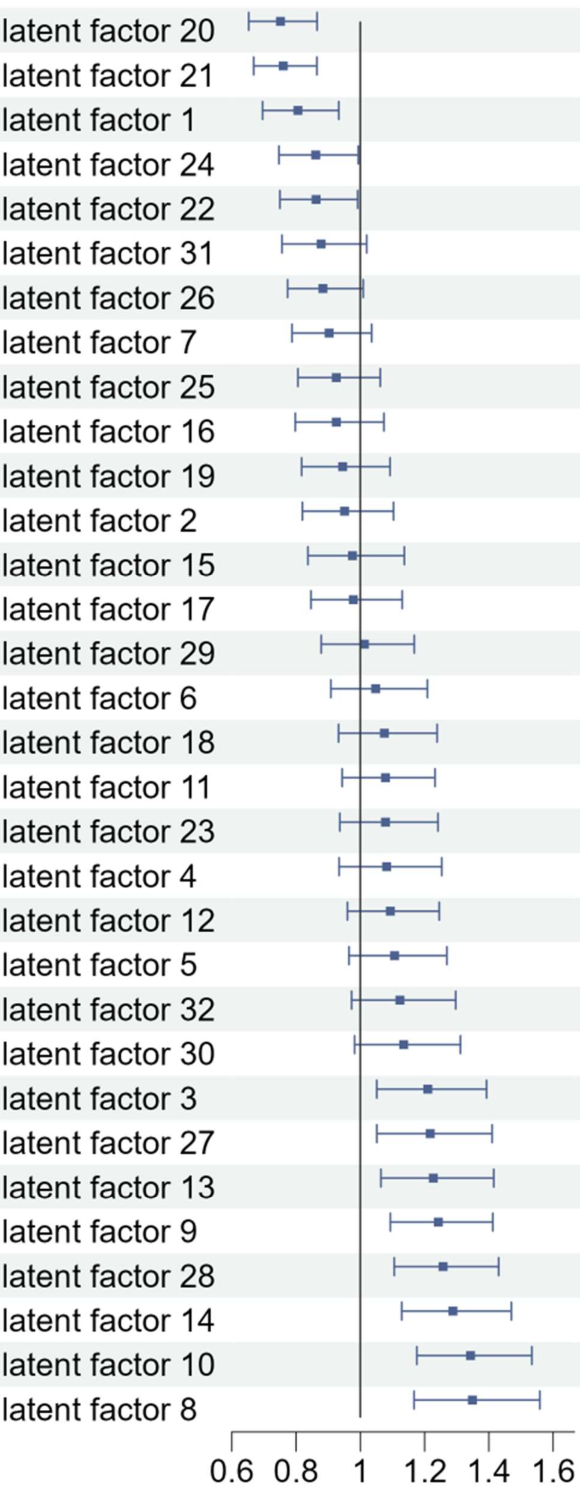

Predictor	HR (95% CI)	p-value
latent factor 20	0.75 (0.65 - 0.87)	7.2e-05
latent factor 21	0.76 (0.67 - 0.86)	3.1e-05
latent factor 1	0.81 (0.70 - 0.93)	0.0039
latent factor 24	0.86 (0.75 - 0.99)	0.04
latent factor 22	0.86 (0.75 - 0.99)	0.038
latent factor 31	0.88 (0.76 - 1.02)	0.088
latent factor 26	0.88 (0.77 - 1.01)	0.067
latent factor 7	0.90 (0.79 - 1.04)	0.14
latent factor 25	0.93 (0.81 - 1.06)	0.27
latent factor 16	0.93 (0.80 - 1.07)	0.31
latent factor 19	0.95 (0.82 - 1.09)	0.45
latent factor 2	0.95 (0.82 - 1.10)	0.51
latent factor 15	0.98 (0.84 - 1.14)	0.75
latent factor 17	0.98 (0.85 - 1.13)	0.76
latent factor 29	1.01 (0.88 - 1.17)	0.86
latent factor 6	1.05 (0.91 - 1.21)	0.52
latent factor 18	1.07 (0.93 - 1.24)	0.32
latent factor 11	1.08 (0.94 - 1.23)	0.27
latent factor 23	1.08 (0.94 - 1.24)	0.3
latent factor 4	1.08 (0.93 - 1.25)	0.29
latent factor 12	1.09 (0.96 - 1.25)	0.18
latent factor 5	1.11 (0.96 - 1.27)	0.15
latent factor 32	1.12 (0.97 - 1.30)	0.11
latent factor 30	1.13 (0.98 - 1.31)	0.086
latent factor 3	1.21 (1.05 - 1.39)	0.0079
latent factor 27	1.22 (1.05 - 1.41)	0.0086
latent factor 13	1.23 (1.06 - 1.42)	0.0049
latent factor 9	1.24 (1.09 - 1.41)	0.00087
latent factor 28	1.26 (1.11 - 1.43)	0.0005
latent factor 14	1.29 (1.13 - 1.47)	0.00017
latent factor 10	1.34 (1.18 - 1.53)	1.4e-05
latent factor 8	1.35 (1.17 - 1.56)	4.9e-05

Figure S6 Forest plot for cardiovascular mortality predicted by traditional ECG parameters

Cardiovascular mortality prediction by the traditional ECG parameters in cox regressions adjusting for age and sex. Hazard ratio (HR) per standard deviation change of the traditional ECG parameters is shown.

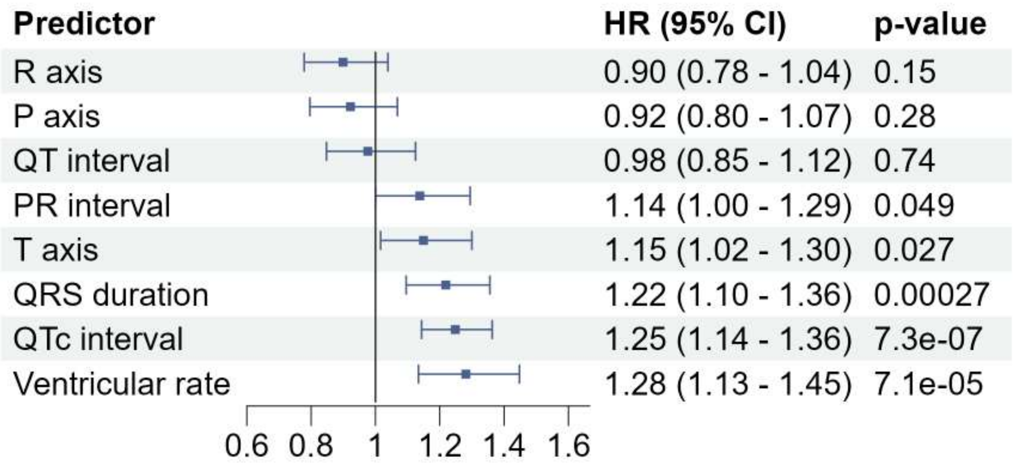
Predictor
HR (95% CI)
p-value
R axis
0.90 (0.78 - 1.04)
0.15
P axis
0.92 (0.80 - 1.07)
0.28
QT interval
0.98 (0.85 - 1.12)
0.74
PR interval
1.14 (1.00 - 1.29)
0.049
T axis
1.15 (1.02 - 1.30)
0.027
QRS duration
1.22 (1.10 - 1.36)
0.00027
QTc interval
1.25 (1.14 - 1.36)
7.3e-07
Ventricular rate
1.28 (1.13 - 1.45)
7.1e-05
0.6
0.8
1
1.2
1.4
1.6

Figure S7 Regional plots of the novel loci identified in the GWAS.

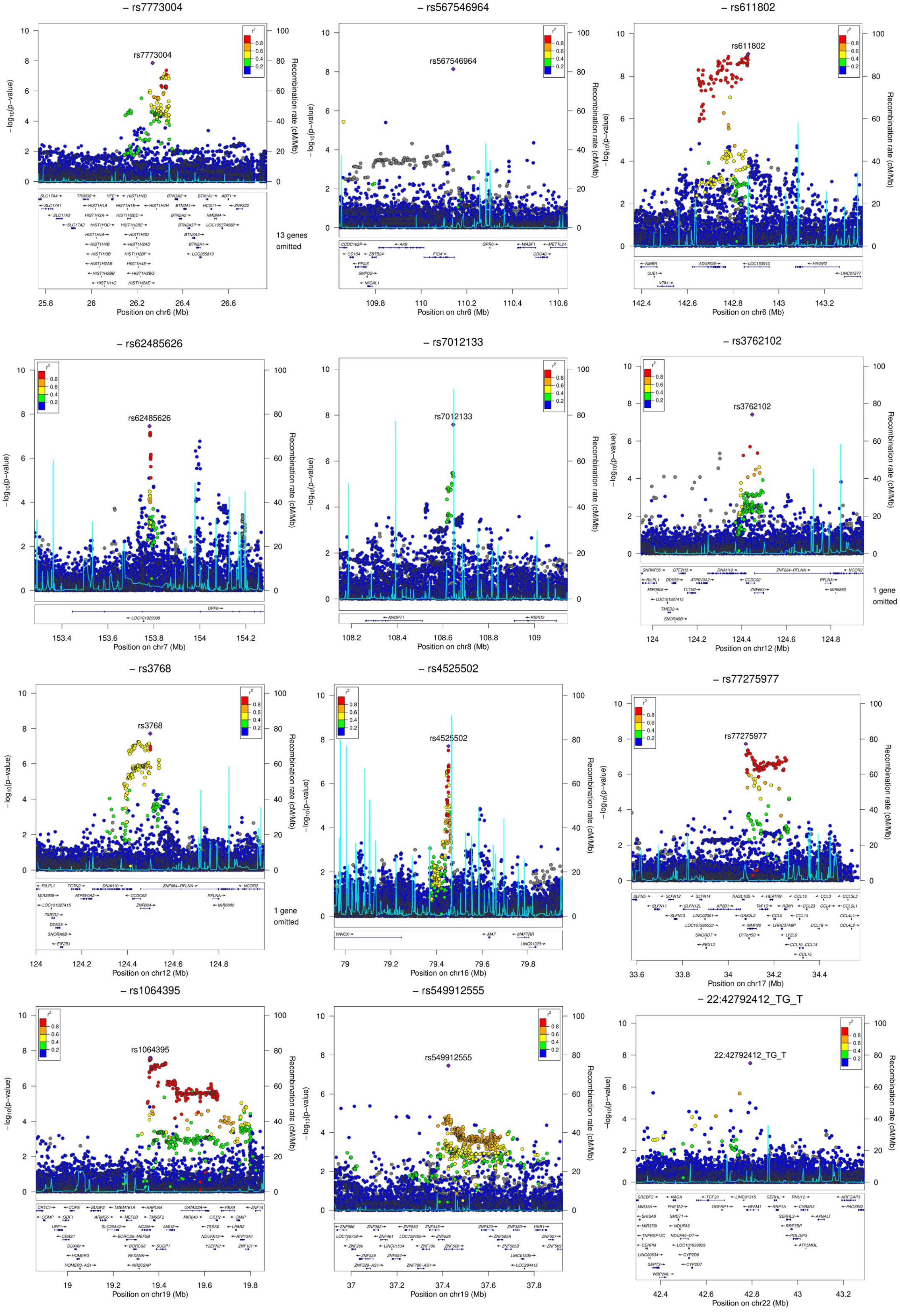

Figure S7 – continued

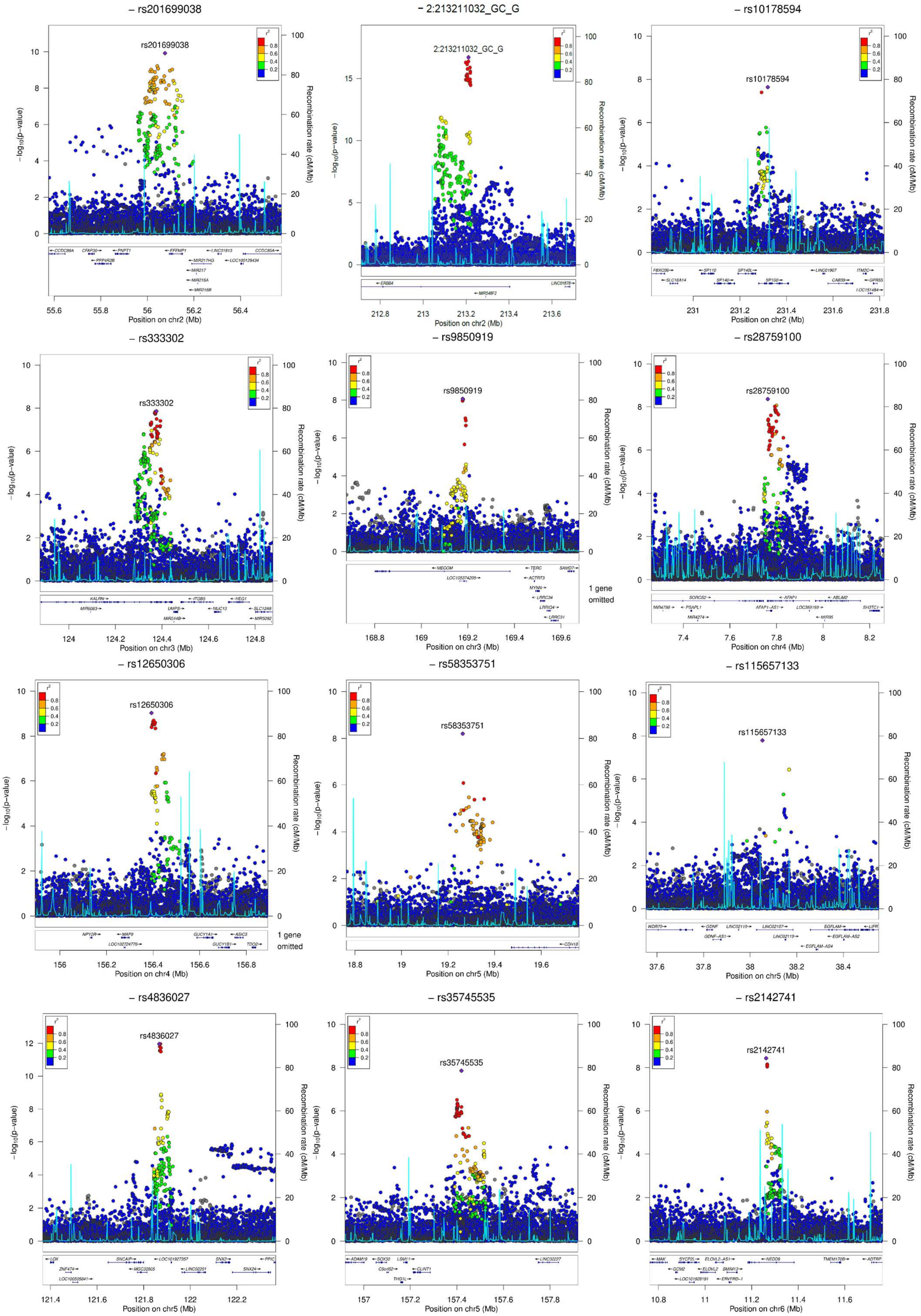

Figure S8 Interactive application for latent factors on ECG morphology

The effect per allele on each dimension for decoded back to the VAE model to visualize the changes in ECG morphology. The app can be accessed at https://genetics.ecgx.ai/.

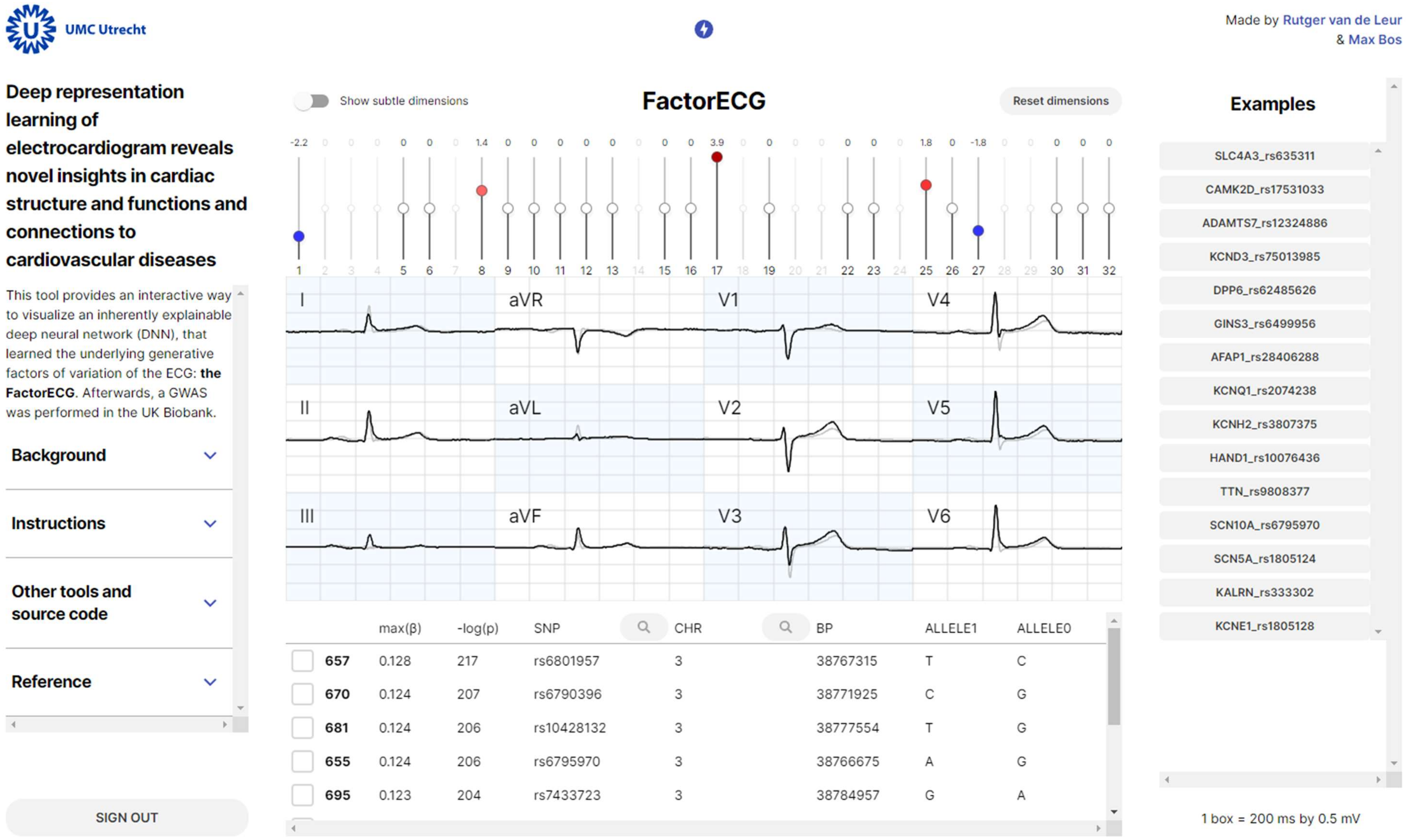

Figure S9 Results from ontology term clustering by semantic similarity

Semantic similarities between the Gene Ontology (GO) associated gene-set by three GO categories, biological processes (BP) , molecula fucntions (MF) and cellular components (CC). Clustering was performed using the binary cut algorithm and results annotated by word cloud of shared words among the gene-sets. Font size reflects the enrichment of the keywords in each cloud.

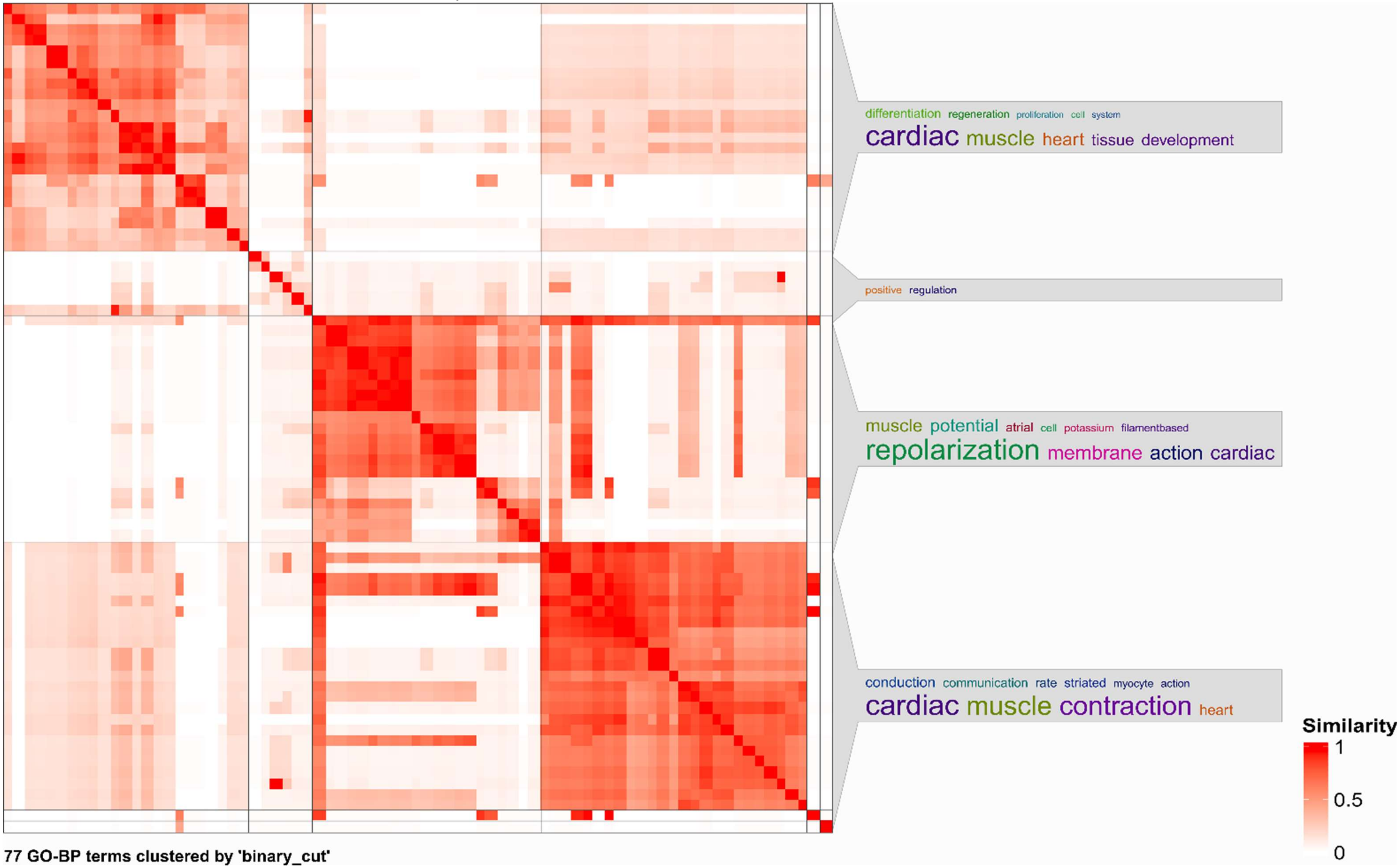

Figure S9 – continued

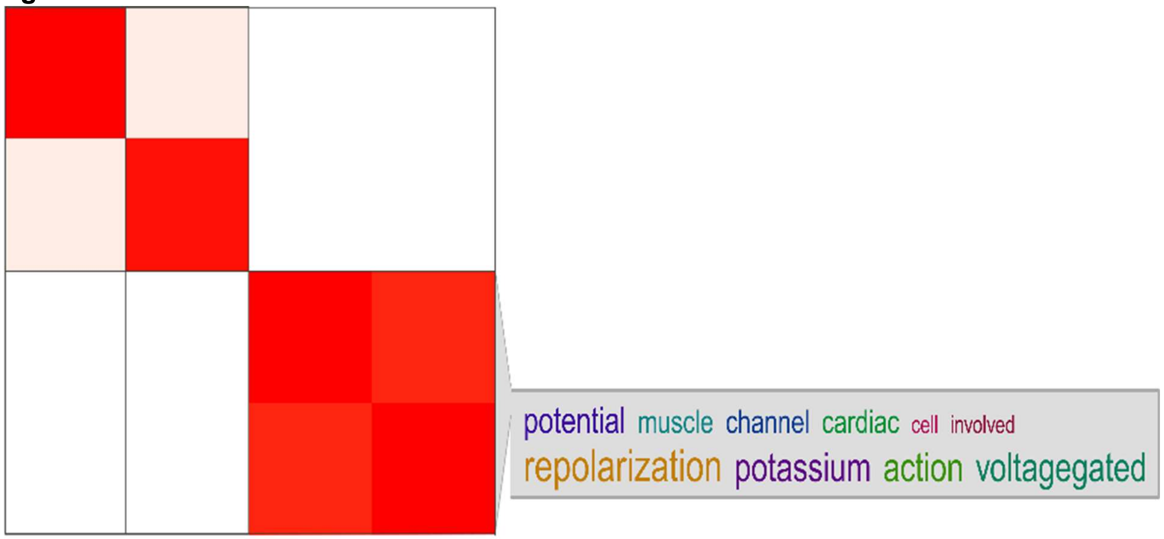

4 GO-MF terms clustered by 'binary_cut'

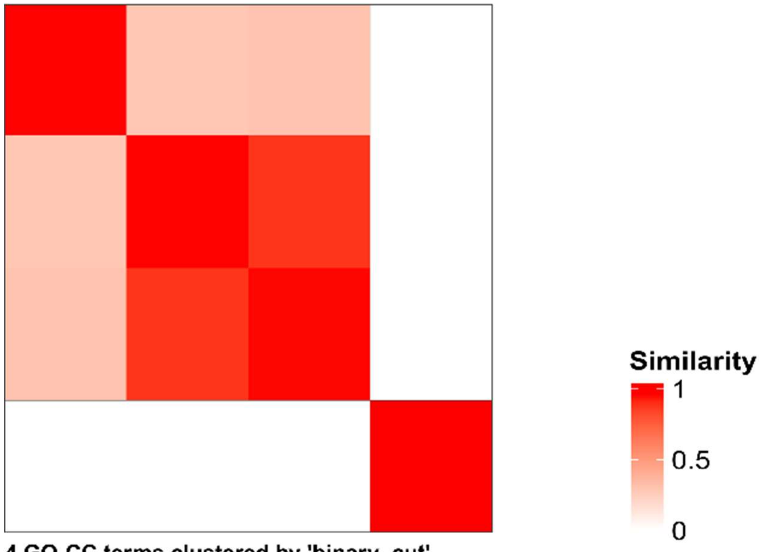

4 GO-CC terms clustered by 'binary_cut'

Figure S10 Genetic correlation of latent factors with cardiometabolic diseases.

Dot plot shows the genetic correlation of the latent factors as a risk factor for selected cardiometabolic disease. The colour of the dot indicates the coefficients in genetic correlations estimated with LD score regression. Coefficients were adjusted for age and sex. The size of dot reflects the p-value categories for the latent factor in the corresponding logistic regression model; $p=8.22 \times 10^{-5}$ is the Bonferroni corrected p-value taking $\alpha=0.05$. The bar plot shows the SNP heritability of the disease or latent factor.

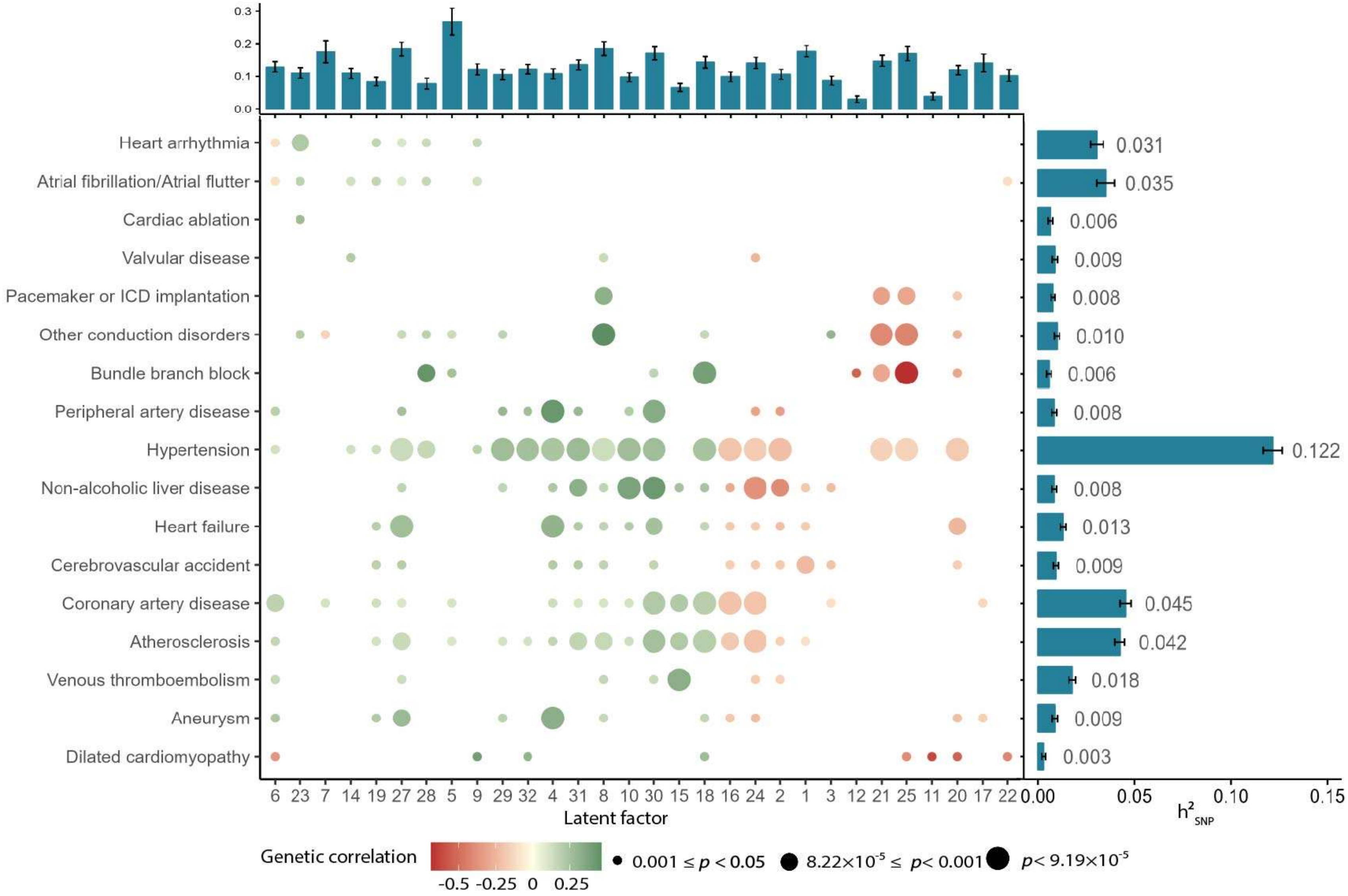

Figure S11 Trait-locus associations reported in GWAS catalog for novel loci.

For each new locus associated with ECG morphology, the lead variant, and all variants in LD>0.8 were queried against the GWAS catalog to identify associations reported in previous GWAS. Each dot represents one association record in the GWAS catalog, coloured by curated biological functional groups.

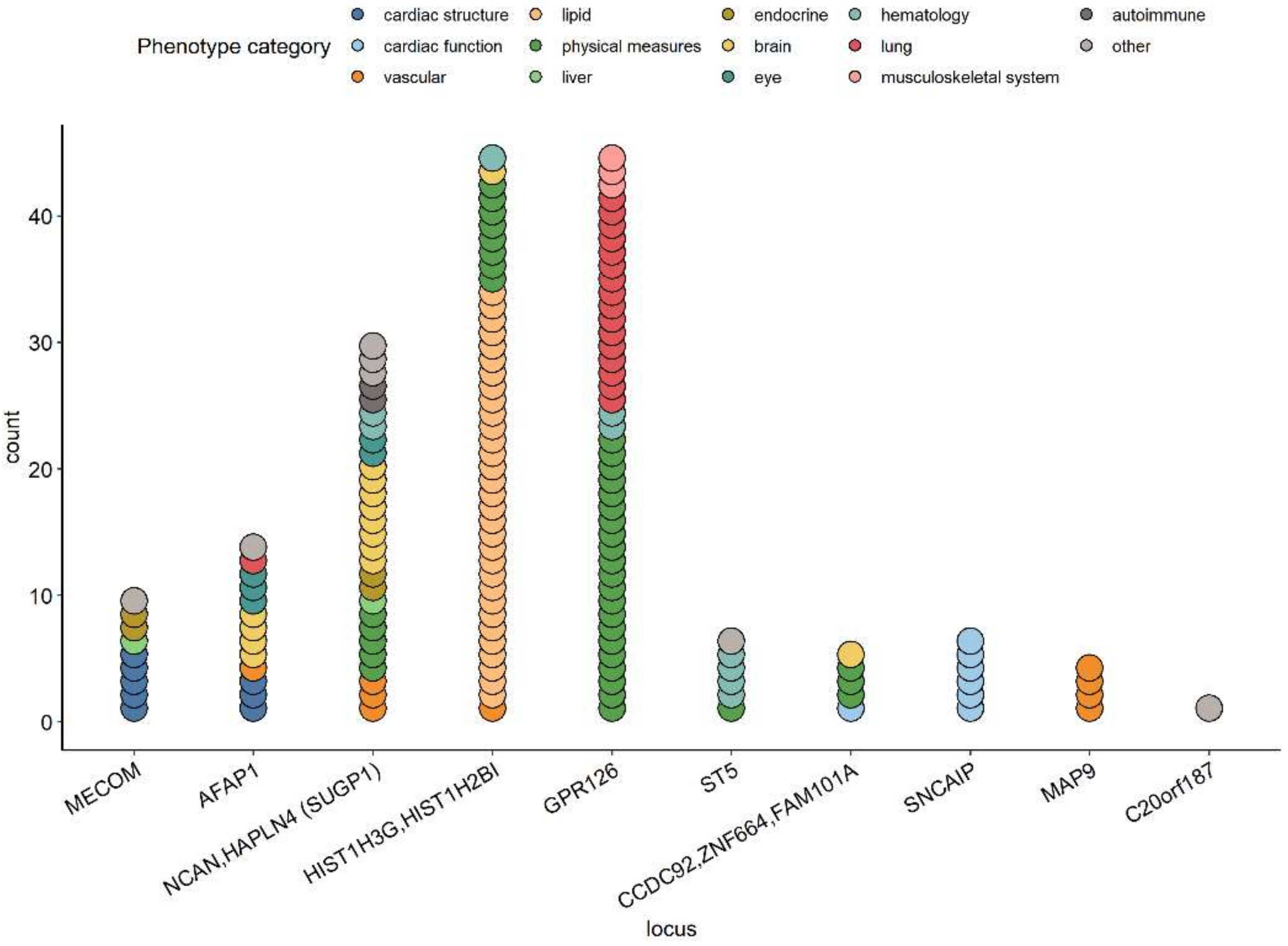

Table S2 Prevalence of diseases at the imaging visit in which the ECG was performed

Outcome	N_{Case}	$N_{Control}$
Aneurysm	6957	439057
Atherosclerosis	71951	374063
Atrial fibrillation/Atrial flutter	27056	418958
Atrio-ventricular block 2nd/3rd degree	3052	442962
Bundle branch block	9760	436254
Cardiac ablation	4346	441668
Cardiac arrest	3143	442871
Cerebrovascular accident	27457	418557
Coronary artery disease	51799	394215
Diabetes	45958	400056
Dilated cardiomyopathy	1415	444599
Heart arrhythmia	39987	406027
Heart failure	16842	429172
Hyperlipidemia	123188	322826
Hypertension	186614	259400
Hypertrohpic cardiomyopathy	645	445369
Non-alcoholic liver disease	7896	438118
Other conduction disorders	16186	429828
Pacemaker or ICD implantation	11340	434674
Peripheral artery disease	10329	435685
Sick sinus syndrome	1241	444773
Valvular disease	17118	428896
Venous thromboembolism	20296	425718
Ventricular arrhythmia	5189	440825
Wolf Parkinson White syndrome	290	445724

Table S3 Clinical characteristics of UKB participants with ECG latent factors generated

Characteristics		Value
N		41927
Age (years), mean (SD)		64.63 (7.73)
Sex women		21621 (51.6%)
BMI (kg/m^2), mean (SD)		26.57 (4.44)
Systolic blood pressure (mm Hg), mean (SD)		134.39 (17.95)
Diastolic lood pressure (mm Hg), mean (SD)		79.29 (8.52)
Ethnicity		
	White	40521 (96.6%)
	Asian	603 (1.4%)
	Black	303 (0.7%)
	Mixed	210 (0.5%)
	Other/Unknown	290 (0.7%)
Current smoker		2978 (7.1%)
Cerebrovascular accident		1107 (2.6%)
Pacemaker or ICD implantation		80 (0.2%)
Heart arrhythmia		1710 (4.1%)
Cardiac arrest		32 (0.1%)
Atrial fibrillation/Atrial flutter		1015 (2.4%)
Ventricular arrhythmia		87 (0.2%)
Sick sinus syndrome		9 (<1%)
Wolf Parkinson White syndrome		44 (0.1%)
Bundle branch block		211 (0.5%)
Atrio-ventricular block 2nd/3rd degree		25 (0.1%)
Cardiac ablation		298 (0.7%)
Other conduction disorders		351 (0.8%)
Valvular disease		696 (1.7%)
Atherosclerosis		3110 (7.4%)
Coronary artery disease		2091 (5.0%)
Heart failure		245 (0.6%)
Dilated cardiomyopathy		24 (0.1%)
Hypertrohpic cardiomyopathy		32 (0.1%)
Peripheral artery disease		408 (1.0%)
Venous thromboembolism		1141 (2.7%)
Aneurysm		196 (0.5%)
Hypertension		14301 (34.1%)
Diabetes		2580 (6.2%)
Hyperlipidemia		12423 (29.7%)
Non-alcoholic liver disease		192 (0.5%)

Table S4 Phenotypic and genetic correlations between the ECG latent factors

Latent factor	Variance	h^2_g	se	$r_g \geq 0.4$
1	0.895	0.209	0.008	NA
2	3.15E-04	0.150	0.008	1,4,12,30,32
3	5.81E-04	0.127	0.008	1,8,21
4	3.44E-04	0.158	0.008	2,12,23,24,27,29
5	0.936	0.278	0.008	NA
6	0.855	0.168	0.008	NA
7	8.97E-04	0.221	0.008	5,14,18,26,29,32
8	0.875	0.239	0.008	NA
9	0.919	0.182	0.008	NA
10	0.850	0.096	0.008	NA
11	0.822	0.044	0.008	NA
12	1.134	0.050	0.008	NA
13	0.907	0.141	0.008	NA
14	7.33E-04	0.127	0.008	7,18,21,28,32
15	0.922	0.108	0.008	NA
16	0.928	0.132	0.008	NA
17	0.855	0.141	0.008	NA
18	2.73E-04	0.191	0.008	5,7,14,25,26,27
19	0.905	0.103	0.008	NA
20	4.54E-04	0.133	0.008	11,12
21	7.15E-04	0.174	0.008	1,3,8,14,28,29,30
22	0.852	0.097	0.008	NA
23	0.823	0.140	0.008	NA
24	5.64E-04	0.187	0.008	4,5,16,30
25	0.944	0.188	0.008	NA
26	0.934	0.201	0.008	NA
27	0.970	0.279	0.008	NA
28	2.91E-04	0.072	0.008	10,14,21,25
29	3.05E-04	0.132	0.008	1,4,7,21,26,31
30	0.968	0.176	0.008	NA
31	0.926	0.206	0.008	NA
32	0.932	0.171	0.008	NA

Table S5 Range of CMR derived structural and functional biomarkers by sex

Parameter	Men	Women	pval
N	20306	21621	
LV end-diastolic volume	84.07 (74.11, 95.11)	75.06 (67.09, 83.47)	<0.001
LV end-systolic volume	28.88 (23.55, 35.04)	23.91 (19.75, 28.55)	<0.001
LV stroke volume	54.59 (47.83, 61.72)	50.69 (45.08, 56.75)	<0.001
LV ejection fraction, %	65.35 (60.90, 69.87)	68.04 (63.81, 72.00)	<0.001
LV end-diastolic mass	55.06 (50.03, 60.67)	44.70 (41.04, 48.90)	<0.001
LV mass-to-volume ratio, g/ml	0.65 (0.59, 0.72)	0.59 (0.55, 0.65)	<0.001
LV peak ejection rate, ml/s	484.74 (412.92, 559.72)	365.67 (306.70, 421.00)	<0.001
LV peak filling rate, ml/s	394.84 (320.74, 471.72)	339.96 (281.07, 399.81)	<0.001
LV mitral annular plane systolic excursion, mm	9.25 (7.96, 10.98)	9.28 (8.10, 10.75)	0.62
RV end-diastolic volume	92.16 (79.64, 106.31)	80.20 (70.53, 90.94)	<0.001
RV end-systolic volume	37.81 (31.92, 44.58)	30.93 (26.12, 36.32)	<0.001
RV stroke volume	54.24 (45.14, 63.67)	49.31 (41.80, 56.80)	<0.001
RV ejection fraction, %	58.73 (54.28, 62.96)	61.36 (56.68, 65.53)	<0.001
RV peak ejection rate, ml/s	447.92 (359.70, 539.42)	341.08 (272.01, 408.55)	<0.001
RV peak filling rate, ml/s	328.84 (252.74, 410.71)	297.75 (236.32, 361.12)	<0.001
RV tricuspid annular plane systolic excursion, mm	13.11 (11.04, 15.51)	12.09 (10.50, 13.87)	<0.001
Heart rate, bpm	60.25 (54.18, 67.48)	62.59 (56.74, 69.42)	<0.001
LV mean myocardial wall thickness, mm	6.22 (5.72, 6.81)	6.23 (5.72, 6.82)	0.88

Table S6 Variance of CMR derived parameters explained by ECG parameters

CMR variable	r^2 age and sex	r^2 age, sex and ECG latent embeddings	r^2 age, sex and ECG parameters
RV tricuspid annular plane systolic excursion	0.0394	0.0633	0.04804
LV mitral annular plane systolic excursion	0.0122	0.0692	0.02097
RV ejection fraction	0.042	0.0884	0.05732
LV ejection fraction	0.0451	0.1399	0.06309
RV peak filling rate	0.1148	0.158	0.14001
RV peak ejection rate,	0.1883	0.2322	0.20931
RV stroke volume	0.0666	0.2469	0.19851
LV peak filling rate	0.2198	0.2618	0.23468
LV mass-to-volume ratio	0.1169	0.2924	0.22085
LV stroke volume	0.0737	0.2961	0.23707
LV end-systolic volume	0.1288	0.3265	0.2288
RV end-systolic volume	0.1897	0.3739	0.32365
LV peak ejection rate	0.3252	0.3765	0.34848
RV end-diastolic volume	0.1561	0.3925	0.33461
LV end-diastolic volume	0.1422	0.4204	0.33054
LV mean myocardial wall thickness	0.3624	0.4701	0.39489
LV end-diastolic mass	0.336	0.4881	0.39496

ECG parameters included: ventricular rate, P interval, QRS duration, QT interval, P axis, R axis and T axis

Table S9 Additional loci identified in MTAG analyses

Analysis	Latent factor	SNP	CHR	hg19	ALLELE1	ALLELE0	A1FREQ	INFO	BETA	SE	pval	Gene	pval < 2.17×10^{-9}	$k_{FINEMAP}$	$prob_k$	SNP with the highest posterier probability
MTAG-1,3,8,14,21,28,29,30	21	rs4245638	1	32349236	G	T	0.772	0.984	0.043	0.008	2.17E-08	*PTP4A2*	0	1	0.786608	rs4245638
MTAG-1,2,4,12,30,32	12	rs79651894	2	38774039	G	T	0.969	0.979	-0.060	0.011	4.48E-08	*HNRNPLL*	0	2	0.541227	rs79651894, rs79773642
MTAG-2,4,12,23,24,27,29	12	rs10769948	11	8761782	A	G	0.535	0.996	0.023	0.004	1.28E-10	*ST5*	1	1	0.826145	rs10769948
MTAG-1,4,7,21,26,29,31	31	rs9556327	13	94441904	C	T	0.703	0.998	-0.041	0.007	4.45E-09	*GPC6*	0	1	0.823468	rs9556327
MTAG-1,4,7,21,26,29,31	26	rs73551879	19	40007654	G	T	0.835	0.960	-0.049	0.009	4.03E-08	*SELENOV*	0	1	0.834536	rs73551879
MTAG-5,7,14,18,26,29,32	26	rs6033054	20	11179048	A	G	0.371	0.998	0.035	0.006	4.73E-08	*C20orf187*	0	1	0.716405	rs6033054

$k_{FINEMAP}$: the number of SNPs of the top causal configuration in FINEMAP; $prob_k$: the posterior probabilities that configurations are the causal configuration by FINEMAP.
